# Cervical Spinal Cord Atrophy Profile in Adult SMN1-Linked SMA

**DOI:** 10.1371/journal.pone.0152439

**Published:** 2016-04-18

**Authors:** Mohamed-Mounir El Mendili, Timothée Lenglet, Tanya Stojkovic, Anthony Behin, Raquel Guimarães-Costa, François Salachas, Vincent Meininger, Gaelle Bruneteau, Nadine Le Forestier, Pascal Laforêt, Stéphane Lehéricy, Habib Benali, Pierre-François Pradat

**Affiliations:** 1 Sorbonne Universités, UPMC Univ Paris 06, CNRS, INSERM, Laboratoire d’Imagerie Biomédicale, F-75013, Paris, France; 2 APHP, Hôpital Pitié-Salpêtriere, Département des Maladies du Système Nerveux, Centre référent SLA, Paris, France; 3 APHP, Hôpital Pitié-Salpêtriere, Service d’Explorations Fonctionnelles, Paris, France; 4 APHP, Centre de Référence Maladies Neuromusculaires Paris-Est, Institut de Myologie, Paris, France; 5 APHP, Hôpital Pitié-Salpêtriere, Service de Neuroradiologie, Paris, France; 6 Sorbonne Universités, UPMC Univ Paris 06, UMR-S975, Inserm U975, CNRS UMR7225, Centre de recherche de l’Institut du Cerveau et de la Moelle épinière–CRICM, Centre de Neuroimagerie de Recherche–CENIR, Paris, France; Inserm, FRANCE

## Abstract

**Purpose:**

The mechanisms underlying the topography of motor deficits in spinal muscular atrophy (SMA) remain unknown. We investigated the profile of spinal cord atrophy (SCA) in SMN1-linked SMA, and its correlation with the topography of muscle weakness.

**Materials and Methods:**

Eighteen SMN1-linked SMA patients type III/V and 18 age/gender-matched healthy volunteers were included. Patients were scored on manual muscle testing and functional scales. Spinal cord was imaged using 3T MRI system. Radial distance (RD) and cord cross-sectional area (CSA) measurements in SMA patients were compared to those in controls and correlated with strength and disability scores.

**Results:**

CSA measurements revealed a significant cord atrophy gradient mainly located between C3 and C6 vertebral levels with a SCA rate ranging from 5.4% to 23% in SMA patients compared to controls. RD was significantly lower in SMA patients compared to controls in the anterior-posterior direction with a maximum along C4 and C5 vertebral levels (*p-values* < 10^−5^). There were no correlations between atrophy measurements, strength and disability scores.

**Conclusions:**

Spinal cord atrophy in adult SMN1-linked SMA predominates in the segments innervating the proximal muscles. Additional factors such as neuromuscular junction or intrinsic skeletal muscle defects may play a role in more complex mechanisms underlying weakness in these patients.

## Introduction

Spinal muscular atrophy (SMA) is a group of degenerative diseases affecting lower motor neurons [1]. SMA diseases are classically separated in proximal and distal forms [1]. The proximal form of SMA is mainly due to deletions or mutations in the telomeric copy of the survival motor neuron gene-1 (SMN1) [2] SMA III is a milder form of SMA starting after the achievement of walking and is subdivided in SMA IIIa with an onset before 3 years and SMA IIIb after 3 years. Eventually, SMA IV refers to an onset in adults (>18 years) [3]. The reason of the classical proximal predominance of the deficit is unknown and its elucidation would provide an important clue to understand the physiopathology of the disease [4]. New developments in spinal cord MRI provide a unique opportunity to investigate *in vivo* cord atrophy and to produce an index reflecting lower motor neurons loss, which has never been reported in any SMA type. In ALS, another motor neuron disease that combines lower and upper motor neuron degeneration, cord atrophy measurement reflected grey matter loss [5, 6]. We tested here the hypothesis that the classical proximal predominance of the motor deficit in SMA may be due to predominant degeneration of lower motor neurons at specific spinal cord levels. We therefore quantified, using spinal MRI and for the first time a surface based morphometry technic, the degree of atrophy at different cervical levels and establish comparisons with the distribution of weakness in SMA patients.

## Material and Methods

### Subjects

SMA patients were recruited from the Myology Institute and the Motor Neuron Disease referral Center (Pitié-Salpêtrière Hospital, Paris, France) between May 2009 and March 2011. Local Ethic Committee approved all experimental procedures (Paris-Ile de France Ethical Committee under the 2009-A00291-56 registration number), and written informed consent was obtained from each participant. All clinical investigations and MRI examinations were conducted according to the principles expressed in the Declaration of Helsinki.

### Clinical examination

Muscle strength was measured by manual muscle testing (MMT) and performed in all patients by the same experienced neurologist (P.-F. P. among the authors) using the Medical Research Council (MRC) scale (0–5) [7]. Particular care was taken to have the patients relaxed before examinations since it was noted that patients with SMA got fatigue easily, which biased muscle strength measurements. MMT subscores were calculated by grouping muscles according to their motor column locations at cervical spinal cord segments: C5 (shoulder abduction), C6 (elbow flexion), C7 (wrist flexion/extension), C8 (abduction of the 1^st^ finger and distal flexion of the 3^rd^ finger). Motor Function Measure (MFM) scale was performed [8]. MFM provides a measurement of the motor capacity by three functional indexes: standing and transfer (D1), axial and proximal motor function (D2) and distal motor function (D3). In addition, SMA patients were scored on the Revised ALS Functional Rating Scale (ALSFRS-R) [9]. An arm ALSFRS-R subscore (handwriting, cutting food and handling utensils), and a leg ALSFRS-R subscore (walking, climbing stairs) were calculated (S1 Dataset).

### MRI acquisition

Subjects were positioned head-first supine, with a 2-centimeter-thick pillow to lift the head and no pillow below the neck. This strategy was used to limit the natural cervical cord lordosis at around C3–C4, i.e., excessive cord curvature in the antero-posterior (A-P) direction. Subjects were positioned as comfortable as possible, centered in the scanner’s reference and were systematically asked not to move during the acquisition in order to minimize motion artifacts.

Scans were performed using a 3T MRI system (TIM Trio 32-channel, Siemens Healthcare, Erlangen, Germany). The spinal cord was imaged using a three-dimensional (3D) T2-weighted turbo spin echo acquisition with a slab-selective excitation pulse (SPACE sequence: sampling perfection with application optimized contrasts using different flip angle evolution). Imaging parameters were: voxel size = 0.9×0.9×0.9 mm^3^; Field of View (FOV) = 280×280 mm^2^; 52 sagittal slices; Repetition time (TR)/ Echo time (TE) = 1500/120ms; flip angle = 140°; generalized autocalibrating partially parallel acquisition (GRAPPA) with acceleration factor R = 3; turbo factor = 69; acquisition time was 6 min [5, 6].

### Data processing

#### Preprocessing

**Fig 1:**
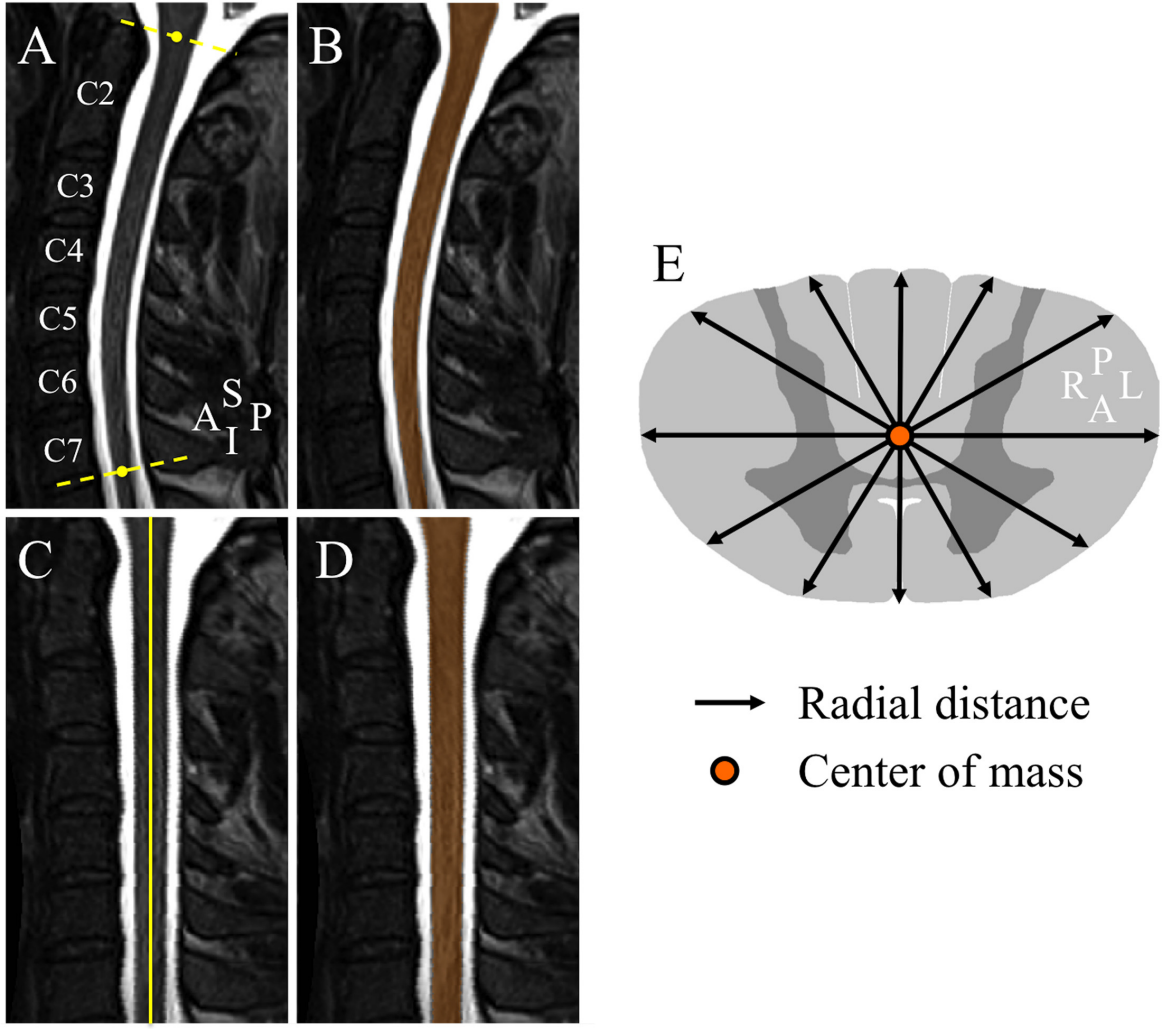
Spinal cord segmentation, cord straightening and radial distance illustration. (A) Preprocessed T2-weighted mid-sagittal section in an SMA patient. The yellow dashed lines delimit the cervical spinal cord region. (B) Resulted segmentation mask (orange). (C) Cervical spinal cord straightening. (D) Mask straightening (orange). (E) Radial distance measurements. A, anterior; I, inferior; L, left, P, posterior, R, right, S, superior.

Data were corrected for non-uniformity intensity using Minc-Toolkit N3 [10]. Two landmarks have been manually defined in the sagittal slice where the spinal cord was the most median in the FOV by an experienced operator on spinal cord MRI segmentation techniques (M.-M. E. M., 5 years of experience, among the authors), to delimit the cervical spinal cord (i.e. from the upper limit of the odontoid process of the C2 vertebrae to the middle vertebral body C7/T1) [11, 12]. Data were cropped and resampled to a voxel size of 0.3×0.3×0.3 mm^3^ using 3D cubic interpolation in order to maximize segmentation accuracy [12–14]. Fig 1A shows a preprocessed mid-sagittal section in an SMA patient.

#### Segmentation

Data were segmented using a double threshold-based method (DTbM), an improved version of the well-established threshold-based method (TbM) [12, 14, 15]. The same experienced operator as previously, adjusted visually rare poor segmentations using TbM and, when needed, manually [12]. Fig 1B shows an example of segmentation result in an SMA patient.

#### Cord standardization

Spinal cord masks resulting from segmentation were straightened, then standardized in length by rescaling them to the median cord length (median length across subjects = 413 slices) using 3D nearest neighbor interpolation, leading to a straight and well centered spinal cord masks in the A-P and right-left (R-L) directions in the FOV [11, 12]. Fig 1C and 1D show an example of cord and mask straightening results in an SMA patient.

#### Radial distance and cross-sectional area measurements

Radiuses from the center of mass of each axial mask to its borders were measured with an angular resolution of 5° by mapping Cartesian coordinates to polar coordinates (one radius every 5°), defined as radial distance (RD) (Fig 1E) [13]. This distance could be seen as a measurement of spinal cord thickness. RD enabled to detect a preferential A-P atrophy direction in spinal cord injuries (SCI) at C2 vertebral level that correlated with the clinical status of SCI patients, represented by the ASIA motor and sensory [13]. RD has also been used to spatially localize the hippocampus diffuse atrophy in Alzheimer patients, a tissue that has a geometry resembling to a bent elliptic cylinder as for the spinal cord [16]. Cross-sectional area (CSA) was also computed. CSA was defined as the cord area in mm^2^ covered by each axial mask along the standardized spinal cord images (S1 Dataset).

### Statistical analysis

Statistical analysis was performed using Matlab^®^ (The Mathworks Inc, MA, USA).

#### Comparison between distal and proximal muscles strength

Wilcoxon signed rank test was performed to test the difference in strength between proximal (MMT C5 spinal level; shoulder abduction) and distal muscles (MMT C8 spinal level; abduction of the 1^st^ finger and distal flexion of the 3^rd^ finger) in SMA patients, a significance level *alpha* = 0.05 was used.

#### Comparisons of MR measurements between patients and controls

RD and CSA along the standardized spinal cord images were compared between SMA patients and controls using permutation test (one side, 100.000 permutations) [16]. Permutation tests measure the distribution of atrophy features represented by RD or CSA decrease in SMA patients compared to controls that would occur by chance if patients and controls were randomly assigned to groups [17]. The high number of permutations 100.000 was chosen to control the standard error of the omnibus probability *p* (SEp) [18]. Given the high number of computed comparisons (72×413 permutation tests for RD comparisons and 413 permutation tests for CSA comparisons), a supra-significance level *alpha* = 10^−3^ was used [13, 16]. The previously constructed template [12] was used as a visualization support for the 3D p-values profile resulted from RD comparisons between SMA patients and controls (S1 Dataset). Mean patients CSA was subtracted from mean controls CSA then normalized by mean controls CSA to obtain cord atrophy rate along the cervical spinal cord (in %).

#### Correlations between MR measurements, clinical disability and disease duration

Spearman's rank correlation coefficient was used to investigate correlations between RD and CSA along the cervical spinal cord and disease duration, MMT, MFM and ALSFRS-R subscores (non-normally distributed and ordinal data). Due to the high number of comparisons (413 correlation tests), a supra-significance level *alpha* = 10^−3^ was used [13, 16]. In addition, CSA gradient from C4 to C7 vertebral levels, corresponding mainly to C5–C8 spinal segments [19] was correlated, using Spearman's rank correlation coefficient, with MMT variation from C5 to C8 spinal segments. Rostro-caudal CSA gradient and proximal-distal muscles strength variation were represented by the slop of the straight line that best fits CSA curve and MMT subscores, resulting from a linear regression model.

## Results

### Demographics and clinical features

Eighteen SMN1-linked SMA patients and 18 age and gender matched healthy volunteers have been recruited. Population demographics and clinical futures in SMA patients are summarized in Table 1. MMT revealed diffuse weakness in the upper limbs involving the muscles innervated by C5 to C8 spinal segments. There was no significant difference between proximal (C5) and distal muscle strength (C8) (Wilcoxon signed rank test; *p*-value = 0.89) in SMA patients.

**Table 1:** Population demographics and clinical features in SMA patients.

Characteristics	SMA patients	Controls
**Number**	18	18
**Age**	36 ± 11 years	35 ± 11 years
**Gender M:F**	10:8	11:7
**Type**	5IIIa, 10IIIb, 3IV	–
**Disease duration**	26 ± 15 years	–
**MMT**		
**Scores**		
**Arm (/70)**	56.3 ± 10.2	–
**Leg (/70)**	46.7 ± 15.2	–
**Total (/140)**	102.9 ± 24.1	–
**C5 spinal level (/10)**	7.3 ± 1.9	–
**C6 spinal level (/10)**	8.4 ± 1.9	–
**C7 spinal level (/30)**	26.1 ± 4.5	–
**C8 spinal level (/20)**	14.4 ± 2.7	–
**Percent changes (%)**		
**C5 spinal level**	73.3 ± 19.4	–
**C6 spinal level**	84.4 ± 18.9	–
**C7 spinal level**	86.9 ± 15.2	–
**C8 spinal level**	72.2 ± 13.5	–
**MFM**		
**D1 (/39)**	19.5 ± 11.1	–
**D2 (/39)**	37.4 ± 1.6	–
**D3 (/39)**	38.3 ± 1.5	–
**ALSFRS-R**		
**Arm (/8)**	7.1 ± 1.2	–
**Leg (/8)**	3.3 ± 1.4	–
**Total (/48)**	40.2 ± 3.8	–

### Study of spinal cord atrophy

#### Comparisons between patients and controls

**Fig 2:**
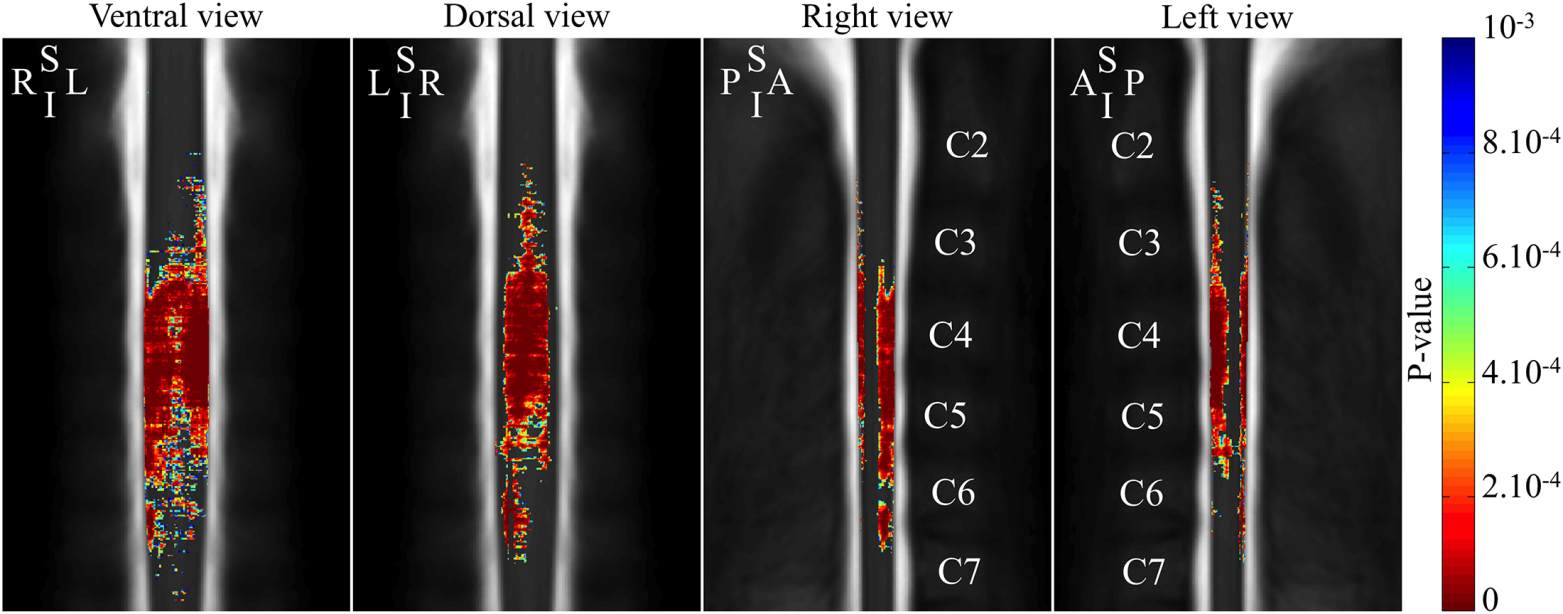
3D atrophy profile along the cervical spinal cord in SMA patients. The color-coding indicates regions with significant atrophy (color scale = p-values of the between groups comparison). A, anterior; I, inferior; L, left, P, posterior, R, right, S, superior.

Fig 2 shows the 3D p-values profile resulting from RD comparisons between SMA patients and controls. RD was significantly lower in SMA patients compared to controls in the anterior-posterior direction and mostly located between C3 and C6 vertebral levels, corresponding to C4–C7 spinal segments, with a maximum along C4 and C5 vertebral levels, corresponding to C5–C6 spinal segments, (*p-values* < 10^−5^) [19].

**Fig 3:**
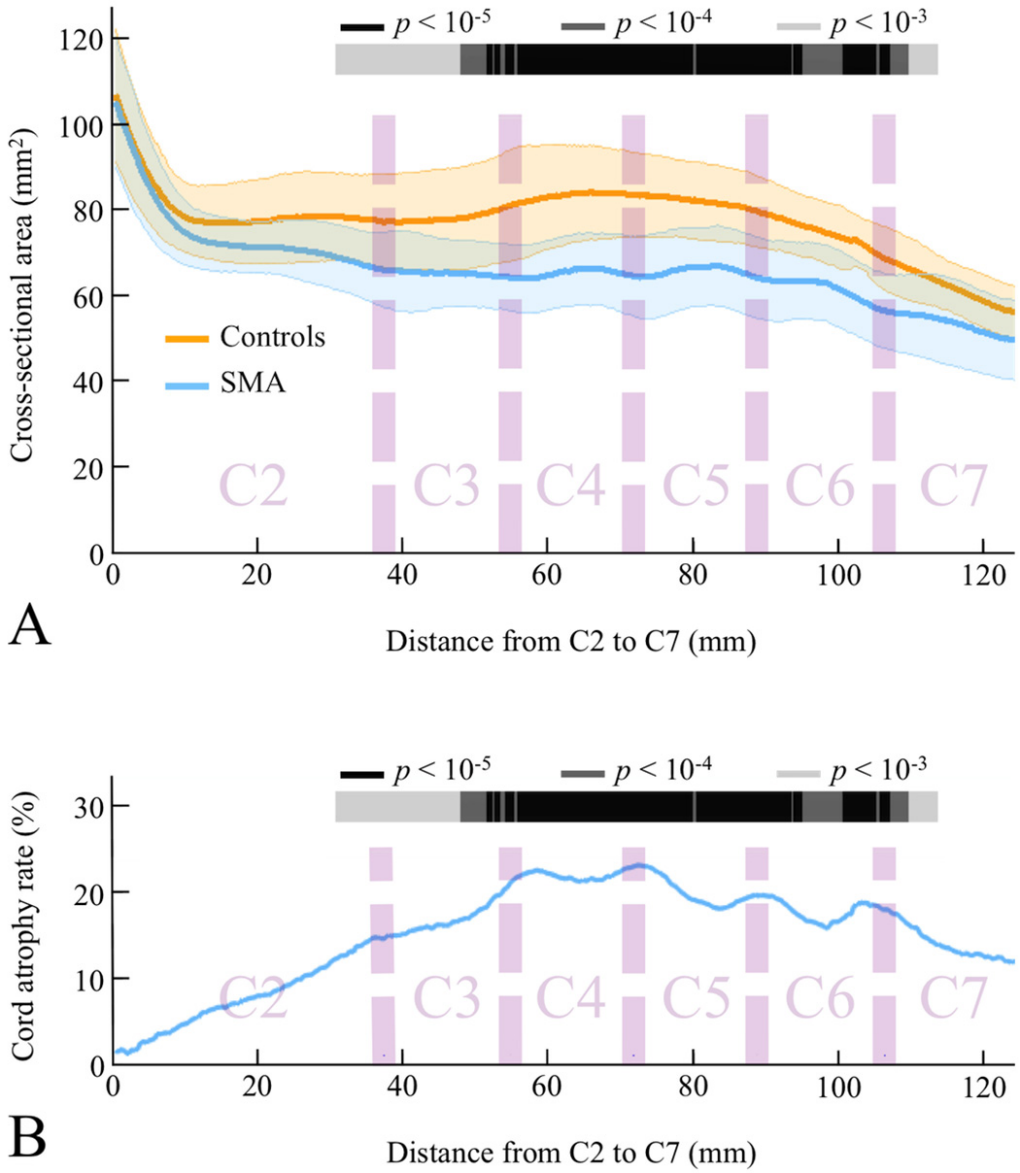
2D atrophy profile along the cervical spinal cord in SMA patients. (A) Cross-sectional area profile in SMA patients and controls. (B) Cord atrophy rate in SMA patients. The color-coding indicates regions with significant atrophy (color scale = p-values of the between groups comparison).

Fig 3A and 3B show CSA profile from SMA patients and controls and cord atrophy rate along the cervical spinal cord, respectively. CSA showed a significant atrophy gradient mainly located between C3 and C6 vertebral levels with a cord atrophy rate ranging from 5.4% to 23%. CSA difference between SMA patients and controls significantly increased from C2 to C3 vertebral level and remained stable along C4 vertebral level with a mean cord atrophy rate equal to 20%. After this plateau, CSA difference decreased slowly and remained significant to the upper part of C7 vertebral level and was no longer significant beyond.

#### Correlations between MR measurements, clinical disability and disease duration

There were no correlations between RD, CSA along the cervical spinal cord and clinical disability subscores MMT, MFM and ALSFRS-R, nor between RD, CSA and disease duration (*p-values* > 10^−3^) (S1–S5 Figs). No correlation was detected between rostro-caudal CSA gradient and proximal-distal muscles strength variation (*p-value* = 0.35).

## Discussion

The main results of the present study are: 1) a three- and two-dimensional cervical spinal cord atrophy profile can be established in SMA types III and IV; 2) the A-P predominance of atrophy suggests that RD, in addition to CSA, could be considered as an index of motor neurons loss in the anterior horns; 3) there were no correlations between RD/CSA and the degree or the topography of clinical deficits.

Spinal cord atrophy was quantified using an accurate segmentation method, DTbM [12]. Segmentation accuracy was increased by adjusting rare poor segmentations using TbM and, when needed, manually [12, 14]. Furthermore, a supra-significance level *alpha* = 10^−3^ was used to minimize false positive results due to error in RD and CSA estimations [13, 16]. The combination of high segmentation accuracy [12] cord standardization procedure that allowed groups’ comparison for large spinal cord segments [11, 12] enabled producing for the first time an accurate 3D cervical spinal cord atrophy profile in SMA patients using RD [12, 16]. RD analysis showed preferential cord atrophy in the A-P direction of the spinal cord between C3 and C6 vertebral levels, corresponding to C4–C7 spinal segments [19]. The apparent atrophy at dorsal side of the spinal cord was probably an indirect consequence of anterior horns atrophy. In fact, the large atrophy in the ventral region produces a displacement of centers of mass of axial masks towards the dorsal side of the spinal cord. Thus, the measured radiuses from the center of mass decreased in both dorsal and ventral regions in SMA patients, which may explain the visible atrophy at both sides of the spinal cord. It cannot also been ruled out that the atrophy of the dorsal column may participate to some extent. Besides post-mortem and histological studies that reported lower motor neuron loss in SMA patients at the cervical cord level [20–22], two autopsy cases of SMN1-linked SMA patients type III showed a loss of the fasciculus gracilis myelinated fibers [22, 23].

The fact that atrophy predominates in the C5 and C6 spinal segments fits with the classical proximal predominance of muscle weakness in SMN1-linked SMA. Our results are also in agreement with a study in delta 7 SMN mice that compared the loss of motor neurons at different spinal cord levels [24]. The authors showed that the reduction of the mean somatic area of motor neurons was maximal at the C5–C6 spinal levels innervating proximal forelimb muscles and was less severe, and not statistically significant rostrally at the C3 level (phrenic motor neurons) and caudally at the C8 level (forelimb distal muscles).

We showed that RD and CSA were not correlated with clinical deficits. First, we did not find correlations between atrophy at a given spinal level and the deficit in the corresponding innervated muscles. Second, the gradient of spinal cord atrophy did not mirror the distribution of weakness in our series of patients. In addition, MMT at the upper limbs showed the presence of distal weakness, which is in accordance with analysis of the literature in SMN1-linked SMA patients type III/IV [25–29]. Interestingly, a study in a rodent SMA model showed that muscle denervation was located throughout the body involving both proximal and distal regions [30].

In our series, the absence of correlation between SCA and weakness could be related to methodological limits, mainly the small number of patients in this rare disease and a lack of sensitivity of MMT for weakness quantification. Despite of these limitations, our findings are in line with the complexity of SMA pathogenesis, which is related to multiple factors, involving motor neuron cell bodies, motor axons and muscles, converging in affecting the integrity and function of the neuromuscular unit leading to muscle deficits [24, 30, 31]. Particularly, studies in various SMA mouse models pointed out that the disease process may be initiated at the neuromuscular junctions with reduced transmitter release, smaller and immature endplates and neurofilament accumulation at presynaptic terminals [23, 32–34]. A recent study using repetitive nerve stimulation showed a decrement in nearly one half of SMA patients types II and III [35]. Such evidence for dysfunction of neuromuscular junction was detected both in distal and proximal muscle groups of the arm and was predominantly postsynaptic. Interestingly, recent findings in different SMA animal models have highlighted the contribution of intrinsic skeletal muscle defects, with disruption of the myogenic program associated with a decrease in myofiber size and an increase in immature myofibers [36]. A study in an SMA feline model provided convincing evidence that motor neuron degeneration begins distal to the cell body and proceeds retrogradely (« dying-back process ») [37].

From a more clinical perspective, the sensitivity of MRI suggests that quantification of spinal cord atrophy could be a potential biomarker of motor neurons loss in SMA, as previously shown in ALS [5, 6, 38]. There is an unmet need for surrogate markers of disease progression in SMA since clinical scales mildly change over time, especially in SMA types III and IV, in which motor disability worsen very slowly [25, 28, 39]. However, longitudinal studies are needed to evaluate if RD and CSA are sensitive to change over time, as shown for CSA in ALS [6] and could be used as a tool to monitor the effect of investigational therapies. These spinal cord MRI metrics are also applicable to other lower motor neuron diseases such as monomelic atrophy (Hirayama disease) to localize local spinal cord flattening and atrophy [40, 41].

Improvement in MRI techniques are promising to increase the sensitivity of the method particularly at the lumbar spinal cord level which remains technically challenging due to increased physiological motions compared to the cervical level [42]. In the future, the development of high-resolution MRI sequences offers the opportunity to specifically measure grey matter loss [42, 43]. However, the absence of correlations between MRI measurements and clinical deficits suggests that the combination with other methods, particularly electrophysiology [35, 44], muscle imaging [44] or electrical impedance myography [45], may be more suitable to measure disease progression in respect with the complexity of the disease.

## Conclusions

This study is a proof of concept that advanced MRI methods can provide indexes of motor neurons loss in the anterior horns in SMN1-linked SMA. Thanks to theses methods, we were able to demonstate that atrophy predominates in the segments innervating the proximal muscles in SMA. In the future, awareness about the potential of advanced MRI methods in this research field should foster collaborative studies that are needed to include larger number of patients and increase the statistical power in transversal and longitudinal studies.

## Supporting Information

S1 Dataset Data set underlying the findings in the present study. MAT-file containing individual MRI data, demographics and clinical features.
(MAT)

S1 Fig 3D correlation profile between MMT C5 subscore and RD along the cervical spinal cord. The color-coding indicates regions with p-values < 0.05 (color scale = p-values of the correlation between MMT C5 subscore and RD).
(TIFF)

S2 Fig 3D correlation profile between MMT C6 subscore and RD along the cervical spinal cord. The color-coding indicates regions with p-values < 0.05 (color scale = p-values of the correlation between MMT C6 subscore and RD).
(TIFF)

S3 Fig 3D correlation profile between MMT C7 subscore and RD along the cervical spinal cord. The color-coding indicates regions with p-values < 0.05 (color scale = p-values of the correlation between MMT C7 subscore and RD).
(TIFF)

S4 Fig 3D correlation profile between MMT C8 subscore and RD along the cervical spinal cord. The color-coding indicates regions with p-values < 0.05 (color scale = p-values of the correlation between MMT C8 subscore and RD).
(TIFF)

S5 Fig 2D correlation profile between MMT C8 subscore and CSA along the cervical spinal cord. The color-coding indicates regions with p-values < 0.05. For correlations between MMT C5–C7 subscores and CSA along the cervical spinal cord, all p-values were higher than 0.05.
(TIFF)
