# Correction: Cervical Spinal Cord Atrophy Profile in Adult SMN1-Linked SMA

**DOI:** 10.1371/journal.pone.0167886

**Published:** 2016-12-01

**Authors:** Mohamed-Mounir El Mendili, Timothée Lenglet, Tanya Stojkovic, Anthony Behin, Raquel Guimarães-Costa, François Salachas, Vincent Meininger, Gaelle Bruneteau, Nadine Le Forestier, Pascal Laforêt, Stéphane Lehéricy, Habib Benali, Pierre-François Pradat

The following information is missing from the Funding section: This study was supported by the Association Française contre les Myopathies-Téléthon (AFM-Téléthon) and the Institut pour la Recherche sur la Moelle épinière et l'Encéphale (IRME). The research leading to these results has also received funding from the program “Investissements d’Avenir” ANR-10-IAIHU-06. The funders had no role in study design, data collection and analysis, decision to publish, or preparation of the manuscript.
