# Supplementary figures and images for: Cervical Spinal Cord Atrophy Profile in Adult SMN1-Linked SMA
Source: PLoS One. 2016 Apr 18;11(4):e0152439. doi: 10.1371/journal.pone.0152439 (PMC4835076; doi:10.1371/journal.pone.0152439)

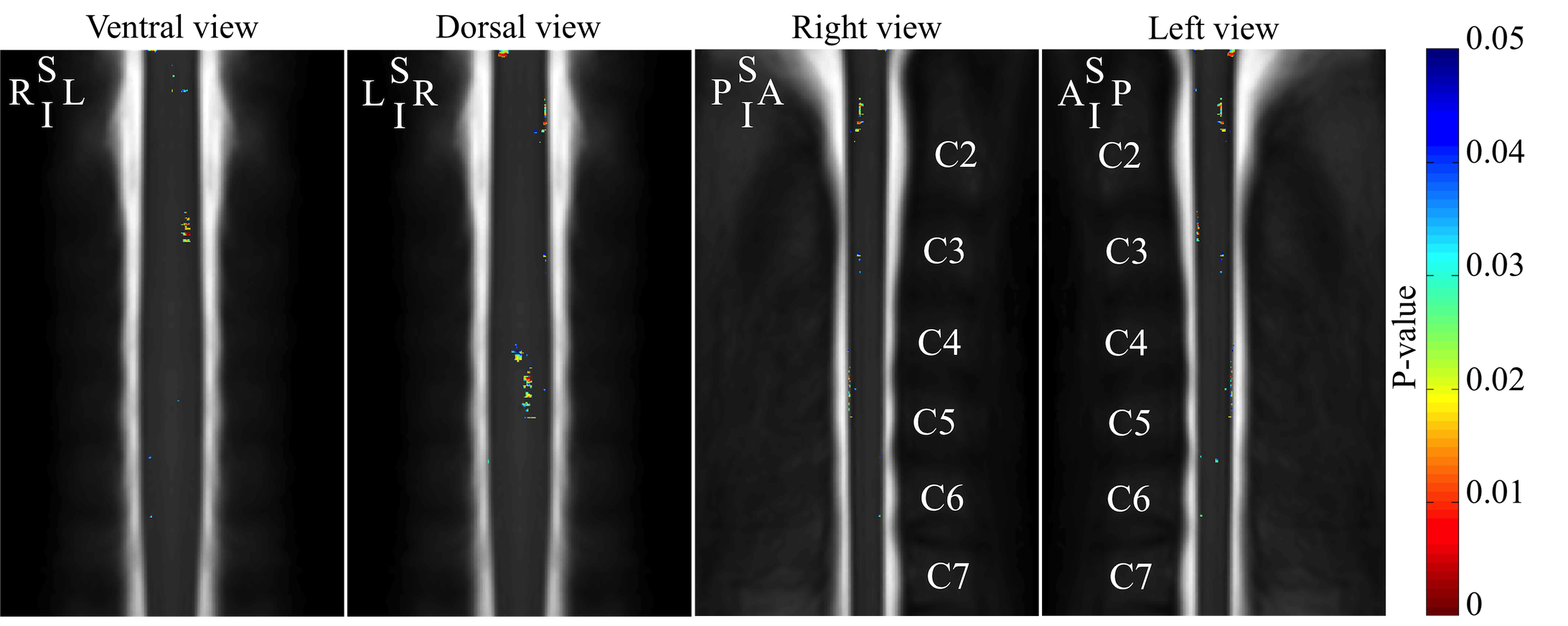

Supplement: S1 Fig — The color-coding indicates regions with p-values < 0.05 (color scale = p-values of the correlation between MMT C5 subscore and RD). (TIFF) [file pone.0152439.s002.tiff]

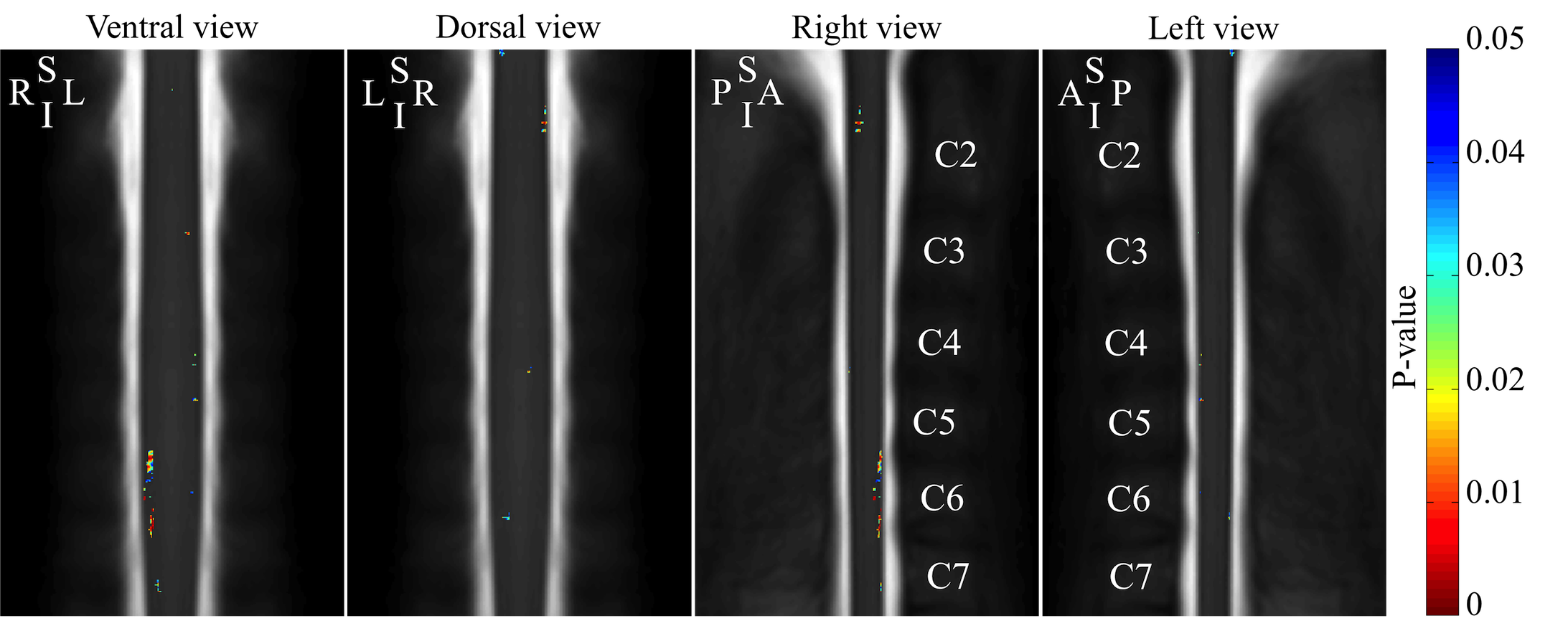

Supplement: S2 Fig — The color-coding indicates regions with p-values < 0.05 (color scale = p-values of the correlation between MMT C6 subscore and RD). (TIFF) [file pone.0152439.s003.tiff]

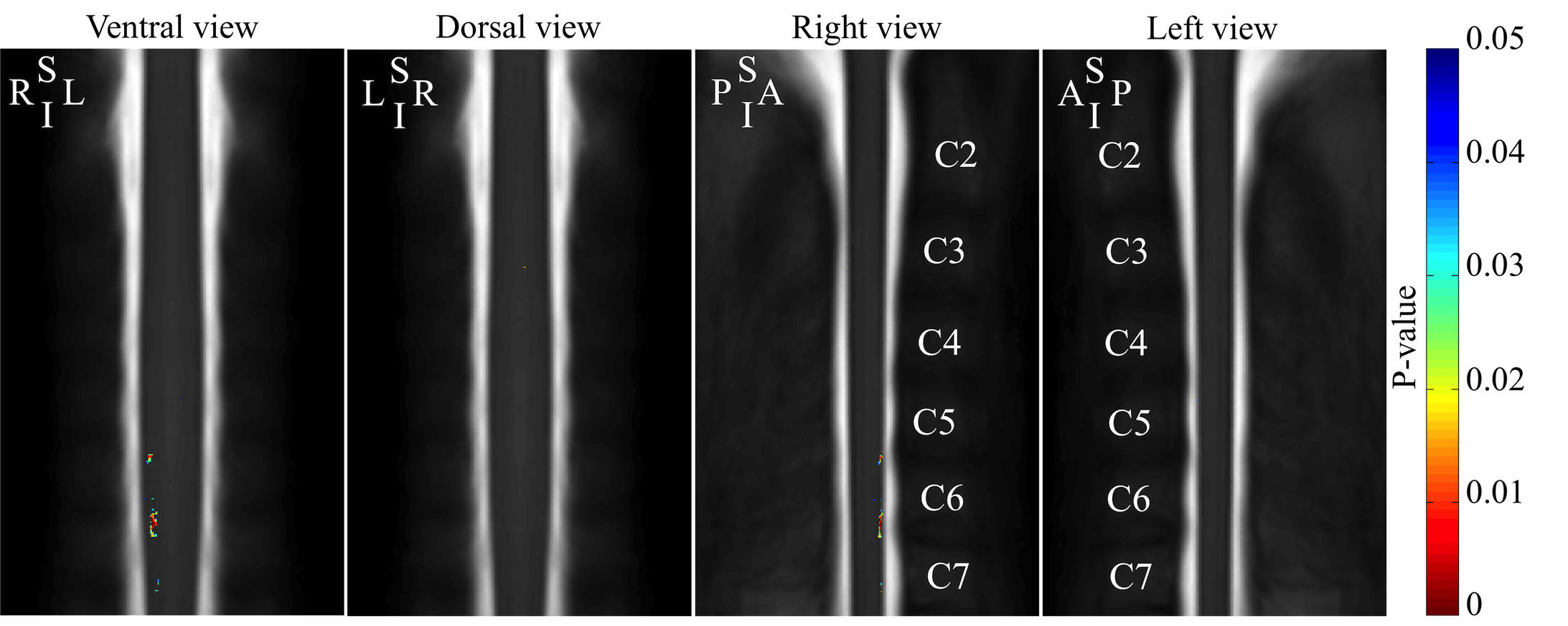

Supplement: S3 Fig — The color-coding indicates regions with p-values < 0.05 (color scale = p-values of the correlation between MMT C7 subscore and RD). (TIFF) [file pone.0152439.s004.tiff]

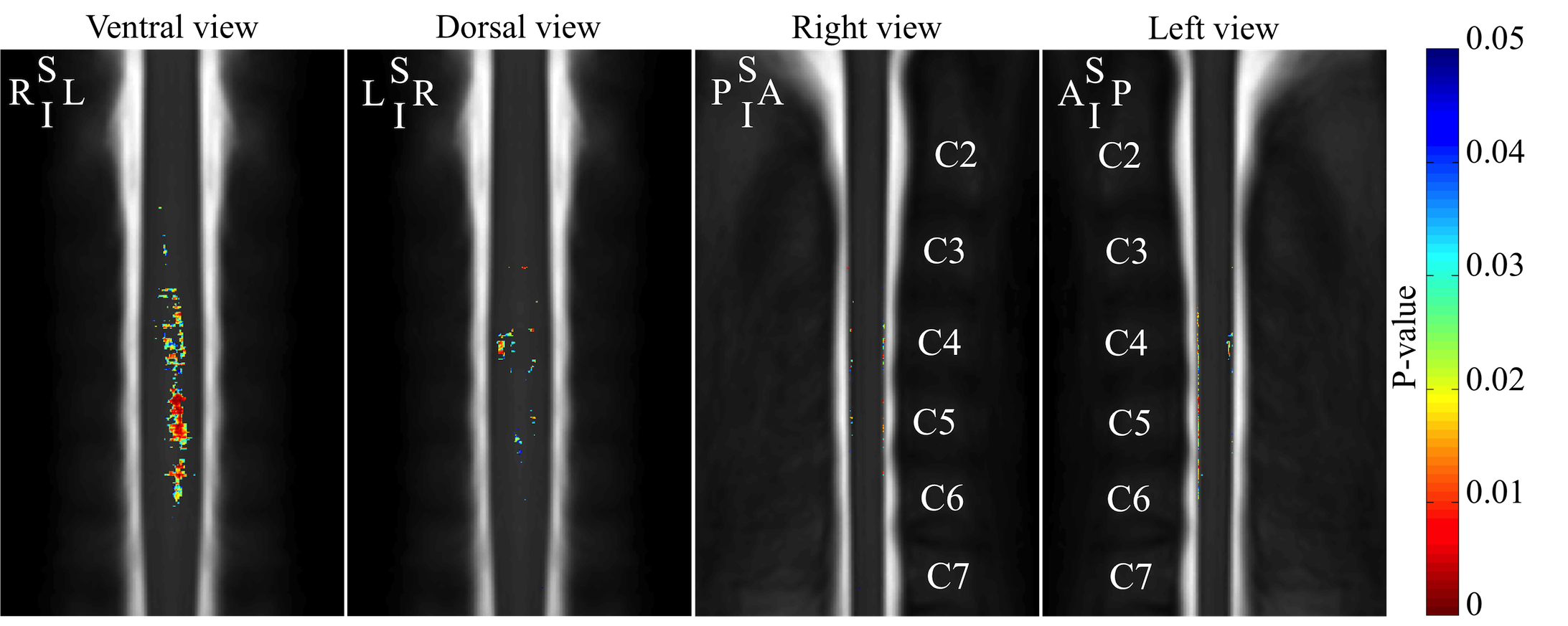

Supplement: S4 Fig — The color-coding indicates regions with p-values < 0.05 (color scale = p-values of the correlation between MMT C8 subscore and RD). (TIFF) [file pone.0152439.s005.tiff]

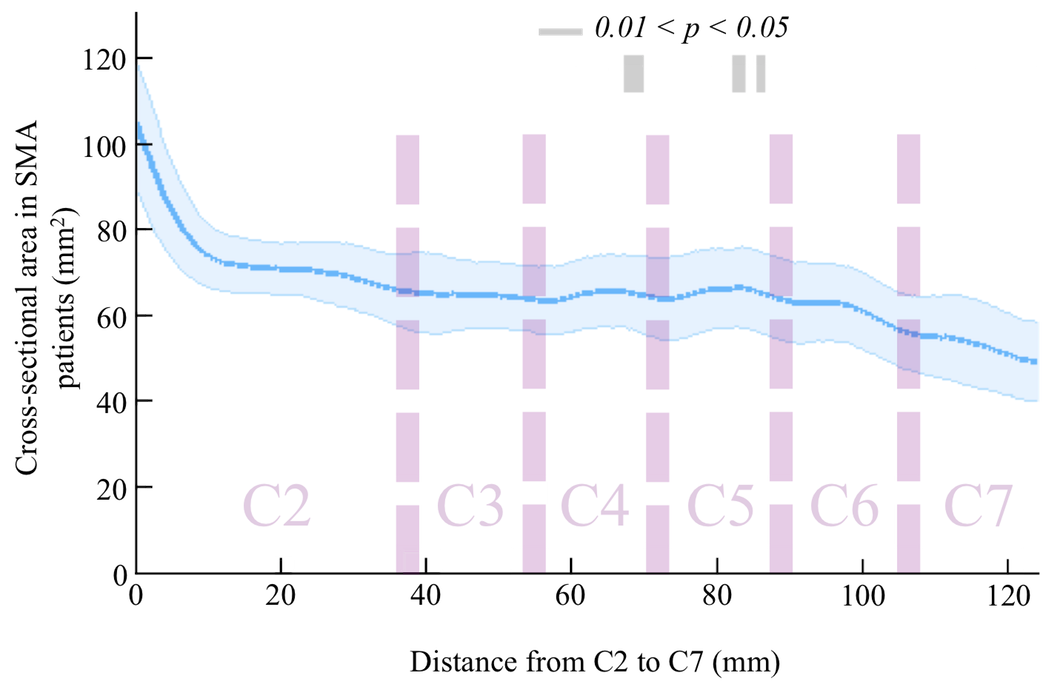

Supplement: S5 Fig — The color-coding indicates regions with p-values < 0.05. For correlations between MMT C5–C7 subscores and CSA along the cervical spinal cord, all p-values were higher than 0.05. (TIFF) [file pone.0152439.s006.tiff]
